# The Peritoneal Cancer Index as a Predictor of Cytoreductive Surgery Outcomes and Heatmapping of Ovarian Cancer Distribution: A Retrospective Analysis

**DOI:** 10.3390/cancers17172790

**Published:** 2025-08-27

**Authors:** Ayisha A. Ashmore, Joud Al-Majali, Samantha Kimi Chui, Susan Addley, Summi Abdul, Viren Asher, Anish Bali, Andrew Phillips

**Affiliations:** 1 Derby Gynaecological Cancer Centre, Department of Gynaecology, University Hospitals of Derby and Burton NHS Foundation Trust (UHDB), Royal Derby Hospital, Uttoxeter Road, Derby DE22 3NE, UK; joud.jboural-majali@nhs.net (J.A.-M.); samantha.chui1@nhs.net (S.K.C.); susan.addley@nhs.net (S.A.); summi.abdul@nhs.net (S.A.); viren.asher1@nhs.net (V.A.); anish.bali@nhs.net (A.B.); andrew.phillips6@nhs.net (A.P.); 2 Royal Derby Hospital, School of Medicine, University of Nottingham, Nottingham NG7 2RD, UK

**Keywords:** ovarian cancer, peritoneal cancer index, cytoreductive surgery

## Abstract

Objective: This study aimed to evaluate the association between the Peritoneal Cancer Index (PCI) and the completeness of cytoreductive surgery (CRS) in patients undergoing surgery for advanced ovarian cancer (AOC). Secondary objectives included identifying a PCI cut-off predictive of incomplete cytoreduction, assessing the relationship between PCI and surgical complexity via the Aletti Surgical Complexity Score (SCS), and exploring disease distribution to better understand ovarian cancer distribution. Methods: A retrospective review of 227 patients undergoing primary or interval debulking surgery for AOC from January 2017 to September 2024 at University Hospitals of Derby and Burton was conducted. PCI was recorded intra-operatively, and procedures were classified using the SCS. ROC analysis identified PCI thresholds for incomplete CRS, logistic regression predicted CRS outcomes, and heat mapping visualised disease distribution. Results: Complete CRS of visible disease (R0) was achieved in 90.75% of patients, while 9.25% had incomplete CRS. Median PCI was significantly higher in incomplete CRS cases (28, IQR 21–32) compared to complete CRS (15, IQR 8–23, *p* < 0.001). ROC analysis identified a PCI threshold of 25.5 with 71.4% sensitivity and 83.5% specificity for predicting incomplete CRS. PCI > 25.5 increased the odds of incomplete cytoreduction by 12.65 times (*p* < 0.001). Higher PCI scores correlated with increased surgical complexity, operative time, and blood loss, though complication rates were similar. Heat maps showed stepwise disease distribution from pelvis to upper abdomen. Conclusions: PCI is a reliable predictor of CRS completeness in AOC, with a threshold of >25.5 indicating a high risk of incomplete cytoreduction. The study underscores PCI’s role in surgical planning and calls for multi-centre studies to validate these findings and further examine disease distribution.

## Simple Summary

This study looked at women with advanced ovarian cancer who had major surgery to remove as much cancer as possible. Surgeons use a scoring system called the Peritoneal Cancer Index (PCI) during surgery to measure how far the cancer has spread within the abdomen. We reviewed the outcomes of 227 patients to see if the PCI score could help predict whether the surgery would be successful in removing all visible cancer. We found that patients with a high PCI score (over 25.5) were much more likely to have incomplete cancer removal. These patients also had longer operations and more blood loss. However, complication rates were similar regardless of PCI score. We also created heatmaps to show where the cancer tends to spread in the body. This information could help doctors plan better treatments and decide how likely it is that surgery will be successful, improving care for women with ovarian cancer.

## 1. Introduction

Standard treatment for advanced ovarian, fallopian tube and peritoneal cancer (AOC) in the United Kingdom (UK) consists of platinum-based chemotherapy and cytoreductive surgery. Outcomes following treatment are improving which has partially been attributed to increasing surgical resection rates and more extensive surgery [1].

Achieving complete resection at cytoreductive surgery (R0) is the most powerful modifiable predictor of survival in advanced ovarian cancer and thus the mainstay of surgery is to remove all visible disease [2,3]. However, with increasing tumour burden the extent of surgery required to achieve complete resection increases, but the intuitive correlation between increasing tumour volume and increased surgical complexity is poorly demonstrated in the literature.

Uncertainty persists regarding the impact of tumour volume and the extent of surgical intervention on achieving R0 resection in ovarian cancer. Beyond these factors, significant gaps remain in our understanding of the natural history of the disease. Historically, ovarian cancer was believed to originate exclusively from the ovaries. However, recent evidence has identified precursor lesions in the fallopian tubes, specifically serous tubal intraepithelial carcinomas (STIC lesions) [4,5,6]. Consequently, it is now recognised that the majority of cancers previously classified as “ovarian” or “peritoneal” are, in fact, of fallopian tube origin.

This shift in understanding has prompted a re-evaluation of the disease’s progression. While it is expected that malignancies originating in the pelvis would disseminate throughout the abdominal and pelvic cavities via transecoelomic seeding, there is limited evidence in the literature to clearly define this progression. Notably, there appear to be two distinct patient populations: one presenting with early-stage, pelvis-confined disease, and another with high-volume, disseminated stage 3C or 4B disease. There is a conspicuous scarcity of cases exhibiting intermediate stages of progression between these groups, suggesting potential gaps in current staging frameworks or diagnostic timelines [7]. Further exploration and research are necessary to clarify these transitional stages and improve our understanding of disease evolution.

As such, two critical questions persist that may be best investigated using an accurate measure of intra-abdominal disease burden. The Peritoneal Cancer Index (PCI) is a well-established tool for assessing tumour burden and distribution within the peritoneal cavity intra-operatively [8]. Originally developed for peritoneal metastases, PCI has been validated across multiple malignancies including colorectal and gastric cancers, both as a means of estimating tumour burden and as a predictor of the likelihood of achieving complete cytoreductive surgery (CRS) [9,10]. In advanced ovarian cancer, PCI similarly offers a robust framework for assessing disease extent and holds promise as a predictive tool for surgical outcomes. Moreover, PCI can facilitate the exploration of the relationship between tumour burden and the extent of surgical intervention, measured in procedural terms such as the Aletti Surgical Complexity Score (SCS) [11].

Additionally, precise mapping of disease distribution enables a better understanding of the likely anatomical spread of disease at various tumour burden, potentially offering insights into the natural history of ovarian cancer.

The aim of this study is to evaluate the association between PCI and the completeness of cytoreductive surgery in patients undergoing surgery for advanced ovarian cancer. Secondary objectives include identifying a PCI cut-off value predictive of incomplete cytoreduction and assessing its impact on surgical outcomes. Furthermore, the study seeks to examine the relationship between PCI as a measure of disease burden and SCS as a measure of surgical complexity. Lastly, the distribution of disease across a comprehensive patient population will be analysed to enhance our understanding of the natural history and progression of ovarian cancer.

## 2. Methods

The study was registered and approved as a retrospective study with the Department of Obstetrics and Gynaecology at University Hospitals of Derby and Burton (UHDB) in accordance with the hospital research and development guidance. All patient data were obtained with permission from the UHDB Caldicott Guardian, ensuring patient confidentiality and compliance with the data protection regulations. Patients were identified from the prospectively recorded hospital databases.

We performed a retrospective review of all patients who underwent primary or interval debulking surgery for advanced ovarian cancer between January 2017 and September 2024. All patients were discussed at a multidisciplinary meeting. Patients were selected for primary surgery or neoadjuvant chemotherapy and interval debulking based on factors such as performance status, serum albumin and presence of ascites, Stage 4A disease at presentation or irresectable stage 4B disease at presentation.

All women undergoing primary or interval debulking cytoreductive surgery for advanced ovarian cancer were included in our study. The study population included patients with International Federation of Gynaecology and Obstetrics (FIGO) stages III–IV. Patients with FIGO stage I–II disease, recurrent disease, and those with no PCI data recorded were excluded. All surgery was performed by appropriately trained gynaecological oncology consultants at an ESGO certified centre for advanced ovarian cancer surgery with prospectively recorded operative findings. All PCI scores were assigned intra-operatively by the same surgical team performing the procedure, ensuring consistency across cases. All histopathology was assessed by specialist gynaecological pathologists.

Surgery was performed with the intention of achieving complete CRS. The PCI score was recorded at the beginning and end of each operation resulting in an initial and final PCI score. For patients undergoing interval debulking surgery, PCI scores were recorded intra-operatively at the time of surgery. Diagnostic laparoscopy was not routinely employed to calculate PCI prior to neoadjuvant chemotherapy (NACT). Each operation was also assigned a surgical complexity score using the Aletti scoring system [11]. This classified each operation into three groups of low, intermediate or high complexity based upon the number and complexity of procedures performed. Surgical data such as duration of surgery, estimated blood loss and complications were collected at the end of each operation. Complications were defined using the Memorial Sloan Kettering Cancer Centre scoring system [12]. Duration of post-operative hospital stay was also collected.

## 3. Statistical Analysis

Statistical analysis was performed using IBM SPSS statistics version 29 and Python (version 3.11; Python Software Foundation, Wilmington, DE, USA) using packages pandas (v2.2.2), numpy (v1.26.4), matplotlib (v3.9.0), seaborn (v0.13.2), and scikit-learn (v1.5.0) [13,14,15,16,17]. Categorical data were summarised using frequencies and percentages. Continuous data were summarised using mean and standard deviation (SD) or median and interquartile range (IQR) depending on normality of distribution. Patient characteristics were compared using Mann–Whitney U test, χ^2^ test or one-way ANOVA test to identify differences between complete CRS and incomplete CRS groups. A *p* value lower than 0.05 was considered statistically significant. Receiver Operator Curve (ROC) analysis was performed to evaluate the ability of initial PCI to distinguish between complete and incomplete CRS. A cut-off for initial PCI for incomplete cytoreduction was also assessed using this method. Binomial logistic regression was also performed to determine whether initial PCI could predict the completeness of CRS. A heatmap of mean initial PCI was correlated with anatomical location. Further multinomial logistic regression was performed to determine whether initial PCI could predict the complexity of surgery. Finally, subgroup analysis was performed using the threshold initial PCI identified from the ROC analysis.

## 4. Results

A total of 227 patients were included in this study between January 2017 and September 2024. The median age of patients included was 66.5 years. 88 (38.8%) underwent primary debulking surgery and 139 patients (61.2%) underwent interval debulking surgery. Two hundred and four patients (89.9%) had serous type ovarian cancer. All other clinical variables and tumour characteristics are described in Table 1.

The median initial PCI for the study population was 16 (IQR 8–23). Complete CRS was achieved in 206 patients (90.75%) and 21 patients had incomplete CRS (9.25%). Patients with incomplete CRS had a higher median PCI of 28 (IQR 21–32) compared with those who had complete CRS (15, IQR 8–23) (*p* < 0.00; Table 1). There were no statistically significant differences in age, FIGO stage, type of primary treatment, histology or complication scores between the complete and incomplete CRS groups. There were statistically significant differences in SCS, mean duration of surgery, mean estimated blood loss and mean duration of hospital stay between patients who had complete and incomplete CRS.

Among patients with FIGO stage 4B disease, complete CRS was achieved in 94 patients (90%). Metastatic lesion locations were frequently located in resectable or chemotherapy-responsive sites as described in Table 2.

### 4.1. Initial PCI as a Diagnostic Test for Completeness of Cytoreduction

**Figure 1:**
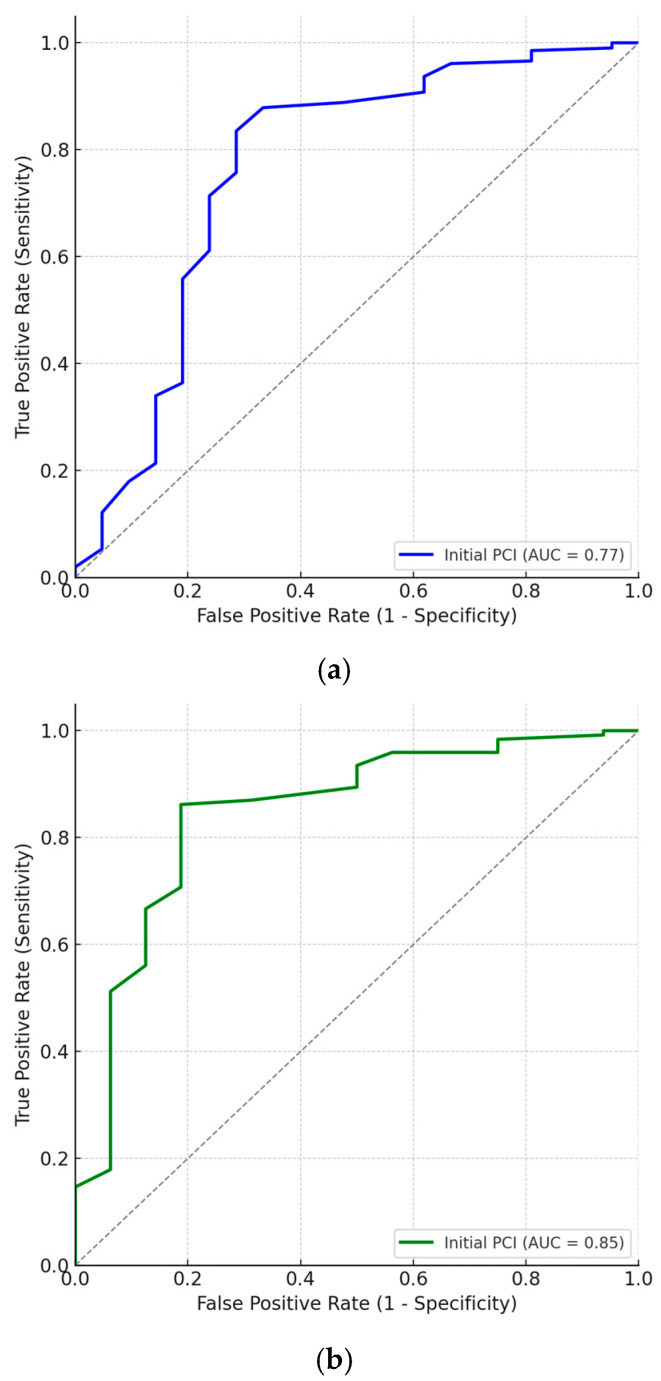
Receiver Operator Curves for initial PCI score and incomplete cytoreductive surgery. This curve illustrates the performance of the initial PCI in predicting incomplete cytoreduction (R1/R2 resection). The diagonal dashed line represents a non-discriminating classifier (AUC = 0.50). (**a**): ROC for all patients undergoing cytoreductive surgery (*n* = 227). The AUC was 0.77, demonstrating good discriminative ability. (**b**): Receiver Operating Characteristic (ROC) curve for patients undergoing interval debulking surgery (IDS) (*n* = 139). The AUC was 0.85, indicating excellent discriminative ability in this post-chemotherapy cohort.

A ROC analysis was then conducted which showed an area under the curve (AUC) of 0.77 (95% CI 0.63–0.90, *p* = 0.00) indicating fair discriminatory ability of the test. The optimal initial PCI threshold value, determined from the coordinates of the ROC was 25.5. Its sensitivity was 71.4% and specificity was 83.5%. When patients undergoing IDS only were considered, initial PCI ROC analysis showed an AUC of 0.85 (95% CI 0.74–0.96, *p* = 0.00). The optimal initial PCI threshold value, determined from the coordinates of the ROC was 26.5 in these patients with a sensitivity of 81.3% and a specificity of 86.2% in correctly classifying patients who had incomplete surgery. Figure 1 demonstrates both ROC.

### 4.2. Initial PCI as Predictor of Cytoreduction

Logistic regression analysis was performed to ascertain the association between initial PCI and cytoreduction. The logistic regression model was statistically significant in our population χ^2^(df 1) = 20.45, *p* < 0.001. The model explained 18.7% (Nagelkerke R^2^) of the variance in patients undergoing incomplete surgery and correctly classified 90.7% of cases. The model predicted that with every 1 unit increase in initial PCI, the odds of incomplete cytoreduction increased by a factor of 1.13. This was statistically significant (*p* < 0.001, 95% CI 1.06–1.19).

When the cut-off threshold of 25.5 was used to predict the odds of incomplete cytoreduction, the logistic regression model was also significant χ^2^(df 1) = 27.13, *p* < 0.001. The model explained 24.5% (Nagelkerke R^2^) of the variance in patients undergoing incomplete surgery and correctly classified 90.7% of cases. The model predicted that having an initial PCI > 25.5 would increase the odds of incomplete cytoreduction by a factor of 12.65 (*p* < 0.001, 95% 4.58–34.92).

### 4.3. Anatomical Distribution of Ovarian Cancer Using Initial PCI

**Figure 2:**
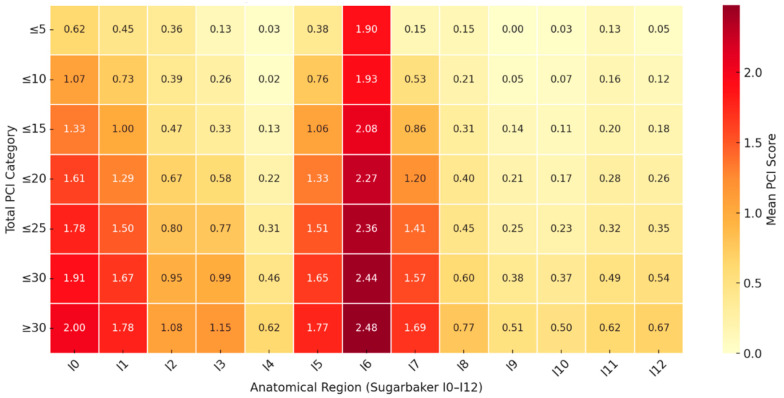
Heatmap showing the mean Peritoneal Cancer Index (PCI) scores by anatomical region (I0–I12) across increasing total PCI categories. Each cell represents the mean regional initial PCI score for patients within a given total PCI range. Regions are based on the Sugarbaker classification (I0–I12), with I6 corresponding to the pelvis. A clear stepwise progression of disease is observed, with early involvement of pelvic (I6) and lower abdominal regions (I5, I7), followed by spread to upper abdomen (I0–I3) and small bowel (I9–I12) as total tumour burden increases.

Figure 2 demonstrates a heat map of initial mean PCI scores within anatomical locations in all patients (Table 3). Tumours expectedly arise in the pelvis (6) and then spread to the lower left and right abdomen (5 and 7) before migrating superiorly to the periumbilical region (0) and then the right upper quadrant (1).

### 4.4. Relationship Between Initial PCI and Surgical Complexity

**Figure 3:**
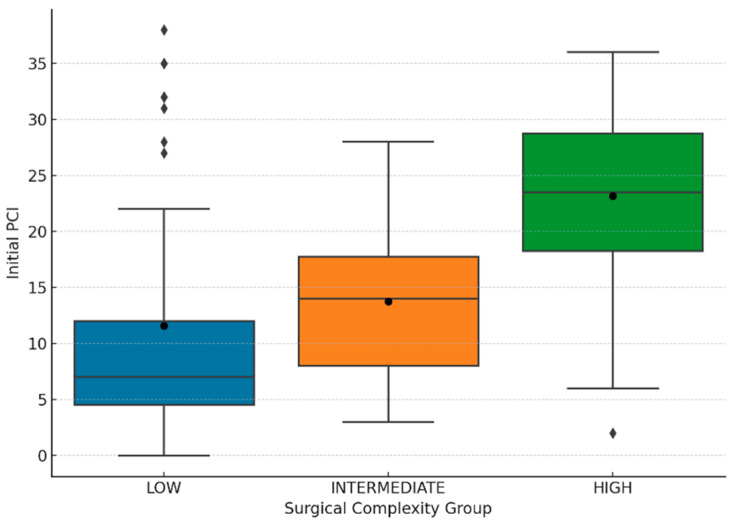
Boxplot of Initial Peritoneal Cancer Index (PCI) stratified by Surgical Complexity Group according to the Aletti score: Low (0–3), Intermediate (4–7), and High (≥8). Boxes represent the interquartile range (IQR), with horizontal lines indicating the median values; whiskers extend to 1.5 × IQR. Black circles denote group means, with mean ± standard deviation shown above each box: Low: 11.6 ± 10.8 (*n* = 55), Intermediate: 13.7 ± 6.3 (*n* = 98), High: 23.2 ± 7.4 (*n* = 74). Rhombi represent outliers beyond 1.5 × IQR. Higher surgical complexity scores were associated with higher Initial PCI values.

Figure 3 shows a statistically significant difference in initial PCI between SCS groups on one-way ANOVA analysis (*p* < 0.00). When subgroup analysis was performed, there was also a statistically significant difference in initial PCI between SCS groups in patients undergoing complete CRS (*p* < 0.00) but this was not the case in those undergoing incomplete CRS (*p* = 0.95).

Linear regression analysis was performed to ascertain the association between initial PCI and SCS groups reflecting surgical complexity. The regression model was statistically significant in our population (B = 11.56, *p* < 0.00). The model explained 28.0% of the variance (R^2^ = 0.28). A high SCS was a significant predictor of initial PCI (*p* < 0.00). On average a patient with a high SCS would have an 11.62 point higher initial PCI score than a patient with a low SCS (*p* < 0.00). An intermediate SCS was not statistically significant in predicting initial PCI when compared with a low SCS (*p* = 0.11).

### 4.5. Relationship Between Initial PCI and Complications of Surgery

There were no statistically significant differences in mean initial PCI scores between complication groups (*p* = 0.34).

### 4.6. Subgroup Analysis of Initial PCI Threshold of 25.5

We used our PCI threshold of 25.5 to perform sub-group analysis. Two groups were formed, one being an initial PCI score of ≤25.5 and the other being initial PCI score of >25.5 (Table 4).

73.47% of patients who had a PCI score of above 25.5 had neoadjuvant chemotherapy whilst the remaining 26.53% of patients had primary debulking surgery. When considering completeness of surgery, in patients with an initial PCI of >25.5, almost 70% of patients had complete CRS compared with 96% of those with an initial PCI of ≤25.5. Additionally, the surgical complexity score was high in 23.03% (*n* = 41) of patients where the initial PCI score was ≤25.5 and 67.3% (*n* = 33) of patients with a score > 25.5. These results were all statistically significant.

Major post-operative complications (Complication score 3–5) occurred in 13.48% of patients with an initial PCI score of ≤25.5 and in 20.41% of patients with an initial PCI of >25.5, however this was not statistically significant.

## 5. Discussion

### 5.1. Main Findings

To our knowledge, we present findings from the largest cohort of patients who have undergone either primary or interval debulking surgery for which intra-operative PCI has been assessed. Our study shows that an initial PCI score of >25.5 has a sensitivity of 71.4% and specificity of 83.5% in accurately predicting incomplete cytoreductive surgery in patients with AOC. Furthermore, in our cohort, having a PCI of over 25.5 meant that a patient was 12.65 times more likely to have incomplete cytoreductive surgery than if the PCI was ≤25.5.

Additionally, we found that a PCI of >25.5 was significantly associated with longer durations of surgery, higher estimated blood losses and longer durations of post-operative hospital stay. Interestingly, there was a significant difference in surgical complexity between PCI groups of ≤25.5 and >25.5. Higher complexity was significantly associated with a lower PCI of ≤25.5 and we suspect this is due to the preponderance of patients with suboptimal, i.e., discontinued surgery in those with a PCI > 25.5. We found no significant difference in complications between PCI score groups. We also assessed the utility of the Aletti SCS in predicting PCI to aid retrospective analysis of cytoreductive operations. We found that a high Aletti SCS could significantly predict initial PCI.

Lastly, disease distribution patterns in ovarian cancer have been described in qualitative terms but visual representations like heat maps are rare. Using mean initial PCI scores, we have developed a heat map showing the natural distribution of ovarian cancer as tumour burden increases. We believe this to be the first study to correlate such findings and graphically show distribution from the pelvis to the upper abdomen prior to invasion of the bowel. This novel visualisation approach may provide a practical tool in understanding tumour spread and aid surgical planning.

### 5.2. Interpretation

Existing studies have shown that PCI is a reliable prognostic tool in ovarian cancer as well as other peritoneal malignancies. Higher scores have consistently been linked to suboptimal debulking and poorer survival outcomes [19,20,21]. Our study corroborates these findings while providing a specific cut-off (initial PCI > 25.5) for predicting incomplete cytoreduction. This PCI threshold is higher than in reported earlier literature but may reflect several key institutional factors [20]. These include variations in surgical aggressiveness, multidisciplinary team decision-making frameworks and differing thresholds for proceeding with surgery based upon disease burden.

Our observed sensitivity (71.4%) and specificity (83.5%) for the PCI cut-off were also slightly lower than those reported in small or more selective cohorts [19,22]. This may reflect the broader inclusion criteria, heterogeneity of tumour biology and real-world complexity of surgical decision-making in a high-volume tertiary setting. Rather than diminishing the utility of PCI, this variation highlights the importance of interpreting thresholds within the context of local operative capability and philosophy.

The absence of a significant difference in complications, despite longer operating times and greater blood loss in patients with complete or incomplete cytoreduction, is noteworthy. This may indicate advancements in perioperative care, patient selection, and surgical expertise. Indeed, previous research has indicated that the number of procedures performed, rather than the execution of certain “high-risk” procedures, more accurately predicts postoperative morbidity [23]. Additionally, we propose that there exist key thresholds where variations in surgery duration or blood loss may be statistically significant but not clinically relevant, or at least not clinically relevant to all patients.

High PCI is often correlated with more extensive disease, which intuitively suggests greater surgical complexity. Previously, surgical complexity has been used to help to describe the extent of surgery performed and to a lesser degree resectability. The SCS has been found to be the only score to significant predict resectability in patients with stage 3 or 4 disease [24]. Our study found that higher surgical complexity was associated with a PCI score of ≤25.5 which could suggest that complete cytoreduction may be more related to surgical effort rather than the extent of disease in agreement with the previous Chéreau et al. findings. Indeed, the findings that high PCI was associated with low SCS in incomplete CRS cases likely reflects aborted or curtailed operations due to unresectability, leading to fewer procedures performed. Thus, SCS may underestimate surgical effort in such contexts and should be interpreted cautiously in relation to PCI. However, despite these limitations our novel evidence showing the predictive value of the SCS as a surrogate for initial PCI may aid retrospective analyses where initial PCI has not been documented or in centres lacking systematic intra-operative PCI assessment, especially in the context of complete resection.

Our heat maps show a clear stepwise progression of disease from the pelvis to other abdominal regions as tumour burden increases. This pattern supports the proposed natural history of OC dissemination via transcoelomic spread. While a large proportion of patients in our cohort received NACT, which is known to reduce visible disease burden at the time of surgery, the spatial distribution of residual disease remained consistent with this anatomical progression. Importantly, prior evidence indicates no significant difference in long-term survival outcomes between patients undergoing primary vs. interval debulking surgery [25], suggesting that while NACT may influence PCI scores, it does not fundamentally alter tumour biology or patterns of spread. Interestingly, recent work has proposed the use of Δ-PCI, the difference between pre-treatment and intraoperative PCI, as a marker of chemotherapeutic response and predictor of resectability. This may help refine selection criteria and better stratify patients for interval surgery [26].

Beyond their contribution to understanding disease progression, the heatmaps also offer practical value to surgeons during operative planning. For instance, if disease is identified intraoperatively in the left upper quadrant, it is reasonable to anticipate corresponding disease in the right upper quadrant. Similarly, disease in the subphrenic spaces may signal a high likelihood of paracolic gutter or central mesenteric involvement. The heatmap thus serves not only as a retrospective tool for understanding tumour dissemination, but also as potentiation intraoperative aid in anticipating areas of disease and ensuring thorough exploration. We propose the integration of such visual tools into clinical pathways may support surgical training, improve intraoperative vigilance and complement radiological or laparoscopic assessments of resectability. Validation of these anatomical distribution patterns in larger multicentre cohorts will be essential to assess their consistency and broader applicability.

### 5.3. Strengths and Limitations

The principal strength of this study lies in the prospective and systematic documentation of PCI scores, despite the retrospective nature of the review. However, the study was conducted at a single ESGO-certified UK cancer centre, and this may affect the generalisability of the findings. Surgical decisions-making thresholds for resectability and intraoperative practices may differ significantly between institutions and health systems. In particular, our centre adopts a maximal-effort surgical approach which may influence both the PCI threshold observed and the rate of complete cytoreduction. Therefore, while our findings are robust within our local context, they should be interpreted with caution elsewhere.

Additional criticisms pertain to the inclusion of both primary debulking surgery (PDS) and interval debulking surgery (IDS) patients. Nevertheless, as previously published, the Derby Gynecological Cancer Centre adheres to a surgical approach aimed at the removal of all abnormal tissue during IDS. Our prior research has demonstrated no significant variation in surgical complexity and a high incidence of malignant disease in areas that might, in other centres, be attributed to “burnt-out disease” in patients who had previously had neoadjuvant chemotherapy [25]. This is consistent with recent findings by Bhatt et al., who reported that macroscopically normal peritoneum after chemotherapy may still harbour residual tumour foci [27]. However, it remains possible that tissue exhibiting a complete response to chemotherapy may have influenced these findings.

Another limitation is associated with patient selection for surgery. Patients with non-resectable stage 4B disease, typically involving hepatic or extensive gastric metastases, may have influenced the results related to region 2, as such patients are less likely to undergo surgical intervention. Furthermore, the unexpectedly low incidence of small bowel lesions warrants consideration. Radiological evidence of small bowel serosal disease is not a criterion for surgical selection, nor do we employ laparoscopic assessments of resectability, preferring instead a maximal effort laparotomic approach. While these findings may result from poor adhesion of disease to mobile abdominopelvic structures, anecdotal observations suggest that several patients exhibited excellent chemotherapeutic responses specifically in the small bowel. Thus, these findings should be interpreted with caution.

Although all patients underwent pre-operative imaging, radiological PCI scoring was not routinely performed and consequently were unable to evaluate the concordance between imaging-based and intra-operative PCI. While this represents a limitation in our dataset, it also highlights a key area for future research. Several studies have shown that CT or MRI can provide a reasonable approximation of surgical PCI and integrating radiological scoring may improve patient selection while also reducing the need for diagnostic surgery [22,28,29]. However, some previous studies regarding resectability based upon imaging have given varying results as surgical ability is expressed in either the cohort inclusion criteria or the operating decisions have likely influence the outcome [30]. Prospective comparison of radiological and surgical PCI in future cohorts could help establish a more streamlined and non-invasive pathway for operative planning.

Validation of these anatomical distribution patterns in larger multicentre cohorts will be essential to assess their consistency and broader applicability. Future research should also explore the integration of radiological PCI scoring to guide operative decision-making and reduce the need for non-therapeutic laparotomy in selected patients. Looking further ahead, artificial intelligence offers opportunities to enhance the predictive power of PCI by combining detailed disease mapping with advanced modelling. Laios et al. recently demonstrated that explainable AI algorithms incorporating anatomical “fingerprints” outperformed PCI scores alone in predicting complete cytoreduction (AUC up to 0.91) [31]. Such tools could, in future, enable real-time, patient-specific surgical planning, complementing operative expertise and optimising cytoreductive outcomes.

## 6. Conclusions

Our study confirms the reliability of initial PCI in predicting the completeness of cytoreductive surgery in advanced cancer. Our cut-off value of >25.5 to predict incomplete surgery was higher than in smaller reported studies but may reflect differences in patient characteristics, surgical skill and unit policies. We support the present assumption of the stage progression of ovarian cancer but questions remain about the biological behaviour of pelvic confined and widespread disease. Finally, we believe that the Alleti SCS has value for historical tumour volume.

## Figures and Tables

**Table 1:** Patient and tumour characteristics in relation to completeness of cytoreductive surgery.

	All Patients (*n* = 227) N (%/IQR)	Complete CRS (*n* = 206) N (%/IQR)	Incomplete CRS (*n* = 21) N (%/IQR)	*p* Value
**Median age**	66.54 (57.43–73.33)	69.95 (57.17–73.08)	65.70 (60.81–74.07)	0.36 *
**FIGO stage**				0.36 **
3A	12 (5.3)	12 (5.82)	0 (0.00)	
3B	13 (5.73)	13 (6.31)	0 (0.00)	
3C	84 (37.00)	75 (36.41)	9 (42.86)	
4A	11 (4.85)	9 (4.37)	2 (9.52)	
4B	104 (45.81)	94 (45.63)	10 (47.62)	
Unknown	3 (1.32)	3 (1.46)	0 (0.00)	
**Primary treatment**				0.14 †
Primary Surgery	88 (38.8)	83 (40.29)	5 (23.81)	
Neoadjuvant Chemotherapy	139 (61.2)	123 (59.71)	16 (76.19)	
**Histology**				0.97 †
Serous	204 (89.87)	185 (89.81)	19 (90.48)	
Non-Serous	22 (9.69)			
Unknown	1 (0.44)	1 (0.49)	0 (0.00)	
**Median PCI**				
Initial	16 (8–23)	15 (8–21)	28 (21–32)	<0.00 *
Final	0 (0–0)	0 (0–0)	24 (9–32)	<0.00 *
**Aletti SCS**				<0.00 **
High	74 (32.60)	70 (33.98)	4 (19.05)	
Intermediate	98 (43.17)	96 (46.60)	2 (9.52)	
Low	55 (24.23)	40 (19.42)	15 (71.43)	
**Median duration of surgery** (minutes)	423 (306–527)	438 (329–537)	225 (140–306)	<0.00 *
**Median estimated blood loss** (millilitres)	700 (400–1200)	700 (450–1200)	350 (150–850)	0.00 *
**Median duration of hospital stay** (days)	12 (8–17)	12 (9–17)	9 (7–14)	0.04 *
**Complication score**				0.59 †
Minor (0–2)	192 (84.58)	175 (84.95)	17 (80.95)	
Major (3–5)	34 (14.98)	30 (14.56)	4 (19.05)	
Unknown	1 (0.44)	1 (0.49)	0 (0.00)	

CRS: Cytoreductive Surgery; IQR: interquartile range; SD: standard deviation; FIGO: International Federation of Gynaecology and Obstetrics; PCI: Peritoneal Cancer Index; * Mann–Whitney U test. ** One-way ANOVA; † χ^2^ test.

**Table 2:** Metastatic lesion locations for patients with FIGO stage 4B disease.

Site of Metastasis	N = 125	%
Paracardiac lymph nodes	56	44.8
Splenic metastases	11	8.8
Hepatic metastases	10	8
Colonic mucosal metastases	7	5.6
Pleural metastases	6	4.8
Supraclavicular lymph nodes	6	4.8
Axillary lymph nodes	6	4.8
Lung metastases	4	3.2
Brain metastases	3	2.4
Hilar lymph nodes	2	1.6
Inguinal lymph nodes	2	1.6
Mediastinal lymph nodes	2	1.6
Cervical lymph nodes	2	1.6
Other	8	6.4

**Table 3:** PCI area and corresponding anatomical location.

PCI Region	Anatomical Location ^a^
0	Periumbilical region (mid-abdomen)
1	Right upper quadrant (right hemidiaphragm, liver surface)
2	Epigastrium (subxiphoid area)
3	Left upper quadrant (left hemidiaphragm, spleen surface)
4	Left flank (left lateral abdominal wall)
5	Left lower abdomen (left iliac fossa, pelvic sidewall)
6	Pelvis
7	Right lower abdomen (right iliac fossa, pelvic sidewall)
8	Right flank (right lateral abdominal wall)
9	Upper jejunum (proximal small bowel)
10	Lower jejunum
11	Upper ileum
12	Lower ileum

^a^ Harmon and Sugarbaker [18].

**Table 4:** Subgroup analysis showing surgical characteristics in relation to initial PCI threshold.

	Initial PCI ≤ 25.5 (*n* = 178) N (%/IQR)	Initial PCI > 25.5 (*n* = 49) N (%/IQR)	*p* Value
**Primary treatment**			
Primary Surgery	75 (42.13)	13 (26.53)	<0.05 †
Neoadjuvant Chemotherapy	103 (57.87)	36 (73.47)	
**Completeness of CRS**			<0.00 †
Complete CRS	172 (96.63)	34 (69.39)	
Incomplete CRS	6 (3.37)	15 (30.61)	
**Aletti SCS**			<0.00 **
Low	45 (25.28)	10 (20.41)	
Intermediate	92 (51.69)	6 (12.24)	
High	41 (23.03)	33 (67.35)	
**Median duration of surgery** (minutes)	411 (306–497)	538 (293–593)	<0.00 *
**Median estimated blood loss** (millilitres)	680 (400–1000)	950 (600–1500)	0.00 *
**Median duration of hospital stay** (days)	11 (8–17)	14 (9–18)	0.12 *
**Complication score**			0.24†
Minor (0–2)	153 (85.96)	39 (79.59)	
Major (3–5)	24 (13.48)	10 (20.41)	
Unknown	1 (0.56)	0 (0.00)	

CRS: Cytoreductive Surgery; IQR: interquartile range; SD: standard deviation; PCI: Peritoneal Cancer Index; * Mann–Whitney U test; ** One-way ANOVA; † χ^2^ test.

## Data Availability

Data available on request.
